# Epidemiological analysis of coronavirus disease (COVID-19) patients on ships arriving at Busan port in Korea, 2020

**DOI:** 10.1371/journal.pone.0288064

**Published:** 2023-07-14

**Authors:** Kee Hun Do, Jinseon Yang, Ok Sook Do, Seok-Ju Yoo

**Affiliations:** 1 Gimhae Airport National Quarantine Station, Gyeongnam Regional Disease Response Center, Korea Disease Control and Prevention Agency, Busan, Korea; 2 Busan National Quarantine Station, Gyeongnam Regional Disease Response Center, Korea Disease Control and Prevention Agency, Busan, Korea; 3 Department of Preventive Medicine, Dongguk University College of Medicine, Gyeongju, Korea; Fiji National University, FIJI

## Abstract

Quarantine played an important role in preventing the spread of infectious diseases between countries in the early stages of the COVID-19 outbreak. In particular, in ports, infection during transit can cause a large number of patients on board ships and can flow into the community. In this study investigated cause of the cause of transmission in ships and suggested the way of preventing secondary transmission by analyzing clinical and epidemiological characteristics of COVID-19 patients identified at Busan Port (South Korea) in 2020. During the study period, out of 19,396 ships that arrived at Busan Port, 50 ships had COVID-19 confirmed cases. Among the 50 ships, type of deep-sea fishing vessels (24 ships, 48.0%), ships weighing less than 5,000 tons (31 ships, 62.0%), and ships from Russia (41 ships, 82.0%) had the highest positivity rates. Total 283 of the 25,450 arrivals tested positive for COVID-19 (a positivity rate of 1.1%), and 270 (95.4%) were asymptomatic. Moreover, the number of COVID-19 patients increased with the duration of the waiting period between arrival and sample collection (12.7% to 37.4%), and the positivity rate was significantly higher for those working as stewards (64.3%). These results indicate secondary transmission was active on board ships and that infection among stewards importantly contributed to group outbreaks. In addition, onboard residence time after arrival significantly elevated to COVID-19 positivity rates, indicating that rapid isolation, as determined using various screening techniques, might be effective at preventing onboard transmission and subsequent community outbreaks.

## Introduction

Coronavirus disease 2019 (COVID-19) rapidly spread worldwide from the outbreak in Wuhan, China in December 2019 [1,2]. COVID-19 exhibits symptoms such as fever and cough, and the virus can be transmitted 1–2 days before the onset of symptoms, which contributes to the risk of group infections [3]. In Korea, on January 20, 2020, the first patient was found at the quarantine stage at Incheon Airport, and the crisis alert was raised to the highest level, ’serious level’, on February 23 to block community transmission [4]. In addition, treatment was provided through early identification and rapid isolation, and special entry procedures were implemented to block overseas inflow [5,6]. Despite the attempts of policy makers, port-borne cluster infections have been continuously reported in ships because individuals live on them for days at a time in crowded conditions. In January 2020, a group infection with COVID-19 occurred on a Japanese cruise ship weighing approximately 110,000 tons, resulting in 705 infections and 6 deaths among 3,711 passengers [7,8]. In Korea, as cases occurred in groups on refrigerated and deep-sea fishing vessels departing from Russia, quarantine policies such as on-board quarantine, COVID-19 testing all disembarking passengers, and submission of a negative PCR certificate upon entry were strengthened to block the inflow into the local community [9–11].

Ports have become increasingly important as entry points of transmission, but few clinical epidemiologic studies have addressed the link between onboard environments and COVID-19 risk. In this study, we analyzed the epidemiological characteristics of COVID-19 patients using COVID-19 diagnostic test data obtained during the quarantine of ships that arrived at Busan Port, a representative port in South Korea. Furthermore, we focused analyzing the first year of the outbreak of COVID-19, 2020. The initial data of the global spread of COVID-19 is relatively free for various variables for analysis of secondary transmission in ships due to low individual immunity differences such as reinfection and vaccination, and low incidence of community infection.

## Materials and methods

### Study design

The COVID-19 test results of all human subjects (25,450) tested for COVID-19 at the Busan National Quarantine Station of the Korea Centers for Disease Control and Prevention Agency (KCDC) that arrived at Busan Port from January 1 to December 31, 2020 were analyzed.

### Data collection

A total of 25,450 sample collection information was provided by the Busan Quarantine Station. Among them, the clinical information of COVID-19 patients (n = 283) was confirmed through the health status questionnaire and epidemiological survey submitted at the time of entry. Information on ships that entered Busan Port, including 50 ships with COVID-19 confirmed cases, used the KCDA Disease and Health Integration System.

### Statistical analysis

The data was coded and transferred to Microsoft Excel (2016) for analysis. COVID-19 positivity rates were analyzed according to gender, age group, type and size of vessel, departure location, and time spent on the vessel using the Statistical Package for the Social Sciences (SPSS) version 20.0 (SPSS, IBM, Armonk, NY, USA).

### Ethics approval

The Institutional Review Board of the KDCA approved the protocol. Consent was waived by the ethics committee (2021-06-02-P-A).

## Results

### 1. Status of COVID-19 tests for arrivals at Busan Port

**Fig 1:**
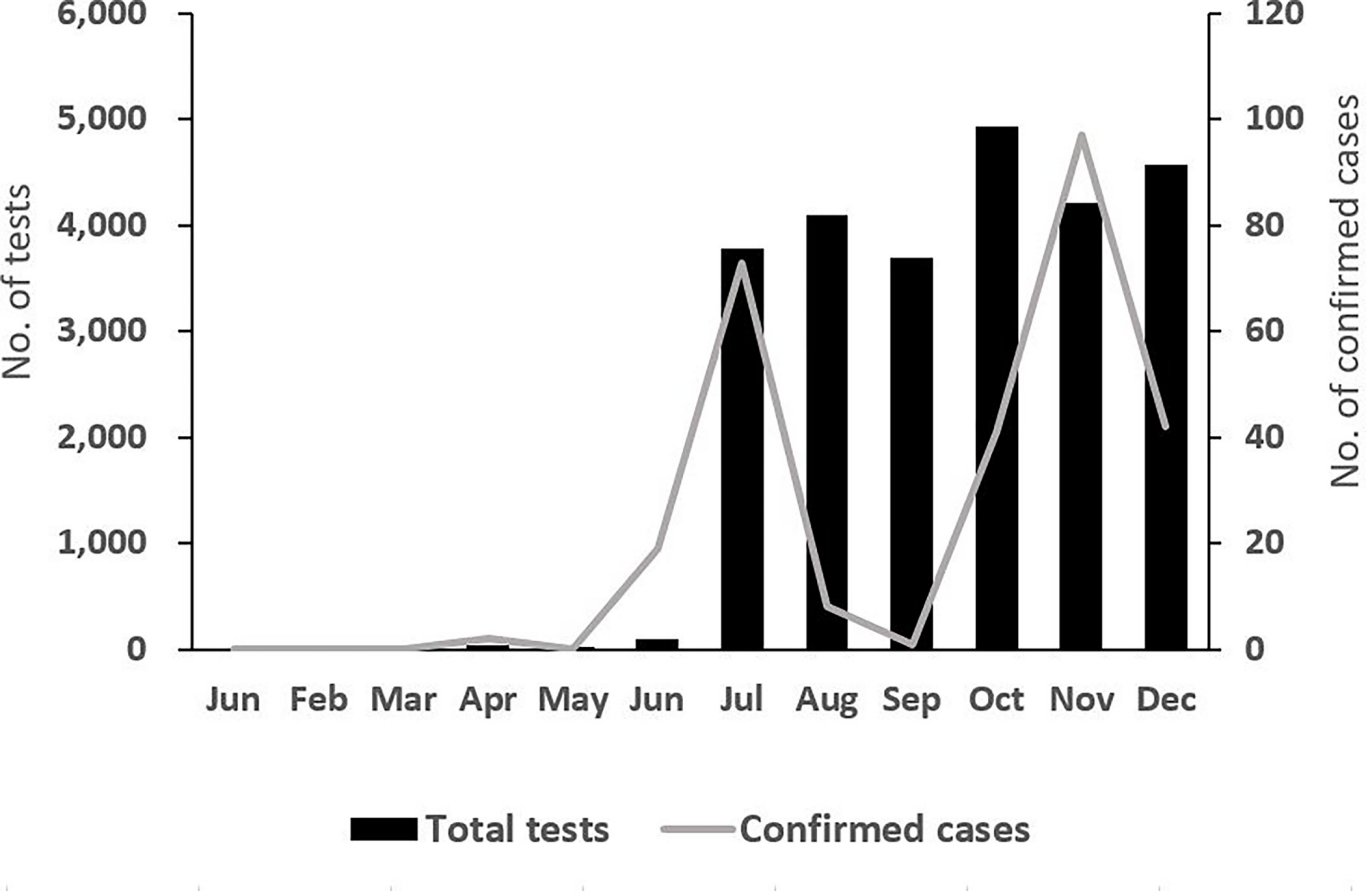
Monthly distribution of COVID-19 testing upon arrival at Busan Port in 2020.

Of the 25,450 subjects tested for COVID-19 in ships that arrived at Busan Port in 2020, 283 (1.1%) were confirmed to have COVID-19. During the year, the number of COVID-19 tests conducted on incoming ships started to increase in June and rapidly increased in July. The number of confirmed cases also increased from 19 in June to 73 in July and 97 in November, which was the largest number of monthly confirmed cases during the investigation period. On the other hand, the COVID-19 positivity rate was highest in June at 19.6% followed by April (5.4%), November (2.3%), July (1.9%), and August (0.2%) (Fig 1).

### 2. General and clinical characteristics of COVID-19 patients in Busan Port

Of the 25,450 subjects, 24,940 were male and 510 were female. Average subject age was 40.9±14.1 years, 6,963 (27.4%) were in their 30s, and Russian nationality was the most common (9,002, 35.4%). The COVID-19 positivity rate was the same for males and females at 1.1%, and was not significantly affected by age (*p* = 0.416). According to nationality, positivity rates were 2.8% for Russian nationals, 0.8% for Filipinos, and 0.0% for other nationalities, and the positivity rate among Russian nationals was significantly higher (Tables 1 and S1).

**Table 1:** Socio-demographic characteristics of COVID-19 cases detected in Busan Port in 2020.

Socio-demographic characteristics	Total tests, no. (%) N = 25,450	Confirmed cases, no. (%) N = 283	Positivity rate, %	*p*-value
**Gender**				
Male	24,940(98.0)	277(97.9)	1.1	0.888 *
Female	510(2.0)	6(2.1)	1.1	
**Age±SD**	40.9±14.1	41.8±8.5	-	
**Age groups(years)**				
Under 20	222(0.9)	4(1.4)	0.0	0.258^†^
20–29	5,970(23.5)	49(17.3)	0.6	
30–39	6,963(27.4)	75(26.5)	1.6	
40–49	5,211(20.5)	73(25.8)	1.7	
50–59	4,223(16.6)	61(21.6)	1.4	
Over 60	2,858(11.2)	21(7.4)	0.8	
Unknown	3(0.0)	-	-	

* Determined by the Chi-square test, ^†^ as determined by the Chi-square test for trend analysis.

Of the 283 confirmed cases at time of entry, 270 (95.4%) were asymptomatic, 9 (3.2%) had fever, 4 (1.4%) had chills, 3 (1.1%) had muscle pain, 2 (0.7%) reported phlegm production, 2 (0.7%) had a cough, 2 (0.7%) had a sore throat, 1 (0.4%) had a headache, and 1 (0.4%) had shortness of breath (Table 2).

**Table 2:** Clinical characteristics of COVID-19 cases detected in Busan Port in 2020.

Clinical characteristics		Confirmed cases, no. N = 283	%
	Asymptomatic*	270	95.4
	Fever	9	3.2
	Chills	4	1.4
	Muscle pain	3	1.1
	Phlegm	2	0.7
	Cough	2	0.7
	Sore throat	2	0.7
	Headache	1	0.4
	Shortness of breath	1	0.4

* Asymptomatic during the quarantine procedure.

※Multiple answers were permitted.

### 3. Characteristics of COVID-19 patient’s staying environment on board

To investigate the characteristics of ships with COVID-19 patients, 50 ships among 19,396 that entered Busan Port during the study period were analyzed. The results obtained showed that deep-sea fishing vessels (3.2%) had the highest positivity rate, followed by frozen refrigerated ships at 1.6%. Furthermore, as regards ship displacements, the positivity rate was highest at 0.6% for ships of < 5,000 tons, and positivity rates decreased with displacement. Among ships arriving from Russia, 41 of 1,533 ships (2.7%) had a COVID-19 positive case, which was higher than the positivity rates of ships that departed from other countries. Moreover, the number of COVID-19 patients that departed from Russia accounted for 232 of the 283 confirmed cases (Table 3).

**Table 3:** Characteristics of ships with COVID-19 patients occurred in Busan Port in 2020.

Characteristics of ships	Ships arrived, no. N = 19,396	Ships with COVID-19 occurred, no. (Cases, no.) N = 50(283)	Positivity rate, %	*p*-value
**Ship type**				
Full container ship	10,877	5(12)	0.0	0.001*
General cargo ship	2,517	3(17)	0.1	
Bulk ship	1,373	1(15)	0.1	
Refrigeration vessel	1,012	16(71)	1.6	
Deep-sea fishing vessel	756	24(167)	3.2	
Oil tanker	27	1(1)	3.7	
Other vessels	2,834	-	-	
**Ship size**				
Under 5,000t	5,193	31(158)	0.6	0.001^†^
5,000t-10,000t	5,335	13(95)	0.2	
Over 10,000t	8,868	6(30)	0.1	
**Departure**				
Japan	7,740	2(17)	0.0	0.001*
China	7,119	2(3)	0.0	
Russia	1,533	41(232)	2.7	
United States of America	635	-	-	
Oceania	533	2(4)	0.4	
Taiwan	293	-	-	
Philippines	176	1(1)	0.6	
Canada	116	1(7)	0.9	
Egypt	5	1(19)	20.0	
Other countries	1,362	-	-	

* Tested by Fisher’s exact test, ^†^ tested by Chi-squared test for trend analysis.

To determine the risk of transmission according to duration on board after departure, we analyzed the positivity rate among 1,500 sailors who boarded ships with COVID-19 patients. Eighty-eight of 306 sailors (28.8%) who remained onboard for > 14 days between departure and sample collection were confirmed to have COVID-19, whereas 195 of 1,194 sailors (16.3%) who remained onboard for < 14 days between departure and sample collection were confirmed to have COVID-19. In the cases of sample collection on day of arrival, the COVID-19 positivity rate was 12.7% but this increased to 37.4% for samples taken at > 3 days after arrival (Table 4). In particular, the positive rate of the steward department sampled 3 days after arrival was 64.3%, which was significantly higher than other departments. (Table 5).

**Table 4:** Influence of staying period on board on COVID-19 positivity rate in Busan Port, South Korea, 2020.

Staying period on board		Total tests, no. (%) N = 1,500	Confirmed cases, no. (%) N = 283	Positivity rate, %	*p*-value
**Departure to sample collection**					
	Within 14 days	1,194(79.6)	195(68.9)	16.3	<0.001*
	After 14 days	306(20.4)	88(31.1)	28.8	
**Arrival to sample collection**					
	Day of arrival	975(65.0)	124(43.8)	12.7	<0.001 ^†^
	1–3 days	322(21.5)	83(29.3)	25.8	
	After 3 days	203(13.5)	76(26.9)	37.4	

* Tested by Chi-squared test, † tested by Chi-squared test for trend analysis.

**Table 5:** Comparison of positive rates by department 3 days after the arrival of a vessel with COVID-19 patients in Busan Port, South Korea, 2020.

Work department		Total tests, no. N = 203	Confirmed cases, no. N = 76	%	*p*-value
**Deck Department**	**Bridge** (Master, Officer)	34	11	32.4	0.045*
	**Deck** (Sailor)	100	33	33.0	
**Engine Department** (Engineer, Motor Man)		51	22	43.1	
**Steward Department** (Cook, Steward)		14	9	64.3	
**Other** (Doctor, Laundress, Etc)		4	1	25.0	

* Tested by Chi-squared test among work departments (Deck, Engineer and Steward department).

## Discussion

As a part of its COVID-19 prevention and control measures, South Korea increased surveillance of inbound travelers to reduce the influx of COVID-19 cases and the risk of transmission by introducing a flexible quarantine management system [12]. In this study, we found a COVID-19 positivity rate of 1.1% (283/25,450) among those arriving by ship in Busan during 2020. The positivity rate was highest among those that departed from Russia (232/283, 82.0%), for ships with displacements of < 5,000 tons (158/283, 55.8%), and for deep-sea fishing boats (167/283, 59.0%). Further, we found that, among confirmed cases, those that worked as stewards and stayed on board for > 3 days after arrival had the highest positivity rate (9/14, 64.3%). This higher positivity rate among those embarking in Russia is in line with the number of cases reported in Russia at the time, for example, 410,000 confirmed cases were reported in June 2020. These results suggest degrees of screening and surveillance be conducted based on considerations of COVID-19 daily reported incidence rates in countries of origin [13,14].

The clinical characteristics of COVID-19 are known to be accompanied by various symptoms, but asymptomatic infections are known to occur at higher rates. At a community treatment center in South Korea, 110 of 330 COVID-19 confirmed cases were asymptomatic, and on the Japanese cruise ship Diamond Princess 17.9% of cases had asymptomatic infections [15,16]. However, in the present study, 95.4% of COVID-19 confirmed cases were asymptomatic at time of entry, which is a substantially higher percentage than has been previously reported. This finding suggests that symptomatic sailors did not board ships or did not report having COVID-19 symptoms. Similarly, foreign arrivals at Incheon International Airport were found to be reticent about reporting their symptoms [17]. These results demonstrate the limitations of symptom-based quarantine and the need for test-based quarantine to identify cases at entry.

The maximum incubation period of COVID-19 is 14 days, and the probability of being COVID-19 positive after 14 days has been reported to be as low as 0.01% [18–20]. In the present study, the number of crew members who stayed on board for more than 14 days was 88 and their positivity rate was 28.8%, which indicated secondary transmissions are common on ships. In addition, most COVID-19 cases occurred on refrigerated and deep-sea fishing vessels, which vessels less than 10,000 tons (data not shown), and duration between arrival and sample collection was associated with higher positivity rates. These results show that the positivity rates in ships increase with occupancy and residence time, support the notion that secondary transmission rates are high onboard ships, and suggest that rapid isolation of confirmed cases is required to block transmission within vessels.

In Busan port, according to the established risk assessment protocol, a sample collection is conducted on all occupants of high-risk vessels on day of arrival, and samples are collected from all those on other vessels during disembarkation. When a confirmed case is found among those disembarking or among symptomatic individuals, all occupants of the vessel are tested, and this process takes about 1 to 3 days after arrival. If a person in contact with a ship is confirmed to have COVID-19 in the community, it takes more than three days for testing. In Korea, there was a case where a repair man that went onboard a ship was infected with COVID-19 by a seaman while working and spread it to the local community [21]. In our study, the positive rate of the steward department sampled 3 days after arrival was 64.3%, which was significantly higher than other departments. These results are presumed to be related to changes in lifestyle patterns on board, such as increased residence time and crowding in restaurants as normal duties terminate on arrival. Steward working areas onboard are narrow, which makes maintaining distances difficult, and densities are high during meals, masks are not worn while eating, and ventilation is poor.

In order to prevent onboard transmission, mealtimes should be staggered, eating individually, and adequate ventilation provided. In addition, test targets should be expanded and COVID-19 cases identified quickly using suitable test methods, such as rapid antigen diagnostic kits and pooled RT-qPCR tests [22–24]. The COVID-19 rapid antigen diagnostic test has lower accuracy and sensitivity than the PCR test, but it can detect 94.1% of asymptomatic infections and results can be obtained quickly.

In summary, it is likely that a proportion of COVID-19 cases found at Busan Port were due to infections contracted in ship restaurants during the waiting period after arrival. To properly address onboard infection, it is important to identify COVID-19 cases promptly after ship arrival using approved testing methods and to institute intensive infection management protocols in restaurants. In particular, test-based quarantine would be expected to reduce onboard transmission substantially and subsequent community transmission.

The present study has several limitations that should be mentioned. In particular, the statistical analysis, the data already collected from the national data network were analyzed as much as possible based on accessible variables, and it is regrettable that multivariate analysis based on more diverse variables could not be performed. Moreover, because data were obtained at time of entry, we were unable to access various factors affecting COVID-19 positivity rate, such as whether health status was checked before boarding, changes in life patterns after arrival, or degree of personal hygiene compliance. Nevertheless, by analyzing the characteristics of COVID-19 cases in ships from various perspectives, we believe that the study provides important basic data for establishing future quarantine policies.

## Supporting information

S1 Table Nationality of COVID-19 cases detected in Busan Port in 2020. (DOCX)

S2 Table Classification of in-ship departments. (DOCX)

S1 Dataset Raw data of COVID-19 patient in Busan Port. (XLSX)
