# Supplementary material for: Epidemiological analysis of coronavirus disease (COVID-19) patients on ships arriving at Busan port in Korea, 2020
Source: PLoS One. 2023 Jul 14;18(7):e0288064. doi: 10.1371/journal.pone.0288064 (PMC10348537; doi:10.1371/journal.pone.0288064)
Supplement: S1 Table — (DOCX) [file pone.0288064.s001.docx]

**S1 Table. Nationality of COVID-19 cases detected in Busan Port in 2020**

| **Nationality** | **Total tests,**  **no. (%)**  **N=25,450** | **Confirmed cases,**  **no. (%)**  **N=283** | **Positivity rate,**  **%** | ***p*-value** |
| --- | --- | --- | --- | --- |
| Russia | 9,002(35.4) | 251(88.7) | 2.8 | 0.001^†^ |
| South Korea | 5,806(22.8) | - | - |  |
| Philippines | 3,554(14.0) | 30(10.6) | 0.8 |  |
| Indonesia | 2,413(9.5) | - | - |  |
| Myanmar | 1,897(7.5) | - | - |  |
| India | 720(2.8) | - | - |  |
| Ukraine | 379(1.5) | 1(0.4) | 0.3 |  |
| China | 362(1.4) | - | - |  |
| Vietnam | 185(0.7) | - | - |  |
| Romania | 125(0.5) | 1(0.4) | 0.8 |  |
| Other countries^*^ | 724(2.8) | - | - |  |

^*^ Sri Lanka, Bangladesh, Taiwan, Thailand, Greece, Poland, USA, Croatia, Ghana, Australia, Germany, Vanuatu, Turkey, Japan, Kirbashi, UK, Malaysia, Montenegro, Nigeria, Denmark, South Africa, Bulgaria, France, Tanzania, Ethiopia, Latvia, Finland, Lithuania, Jordan, Belarus, Netherlands, Egypt, Georgia, Morocco, Estonia, Pakistan, Italy, New Zealand, Israel, Panama, Hong Kong, Cypress, Singapore, Serbia, Azerbaijan, Uzbekistan, Canada, Chile, Papua New Guinea, Iraq, Peru, Brazil, Africa, Bosnia-Herzegovina, Fiji, ^†^determined by the Chi-square test
