# Supplementary material for: Epidemiological analysis of coronavirus disease (COVID-19) patients on ships arriving at Busan port in Korea, 2020
Source: PLoS One. 2023 Jul 14;18(7):e0288064. doi: 10.1371/journal.pone.0288064 (PMC10348537; doi:10.1371/journal.pone.0288064)
Supplement: S2 Table — (DOCX) [file pone.0288064.s002.docx]

**S2 Table. Classification of in-ship departments.** The department on board are classified based on the typical place of work.

| Work department | | Position on board |
| --- | --- | --- |
| Deck Department | **Bridge** | Master, Chief Officer, 2nd Officer, 3rd Officer, Radio Electronic Officer |
|  | **Deck** | Boatswain, Sailor, Fisher man, Seamen |
| Engine Department | | Chief Engineer,1st Assistant Engineer, 2nd Assistant Engineer, 3rd Assistant Engineer, 4th Assistant Engineer, Motor Man, Electrician, Oiler/Wiper, fitter |
| Steward Department | | Chief Steward, Cook |
| Other | | Doctor, Laundress, Cleaner |
